# Molecular Hydrogen Prevents Osteoclast Activation in a Glucocorticoid-Induced Osteoporosis Zebrafish Scale Model

**DOI:** 10.3390/antiox12020345

**Published:** 2023-02-01

**Authors:** Marta Carnovali, Giuseppe Banfi, Massimo Mariotti

**Affiliations:** 1 IRCCS Orthopedic Institute Galeazzi, Via C. Belgioioso 173, 20161 Milan, Italy; 2 School of Medicine, Vita-Salute San Raffaele University, Via Olgettina 58, 20132 Milan, Italy; 3 Department of Biomedical, Surgical and Dental Sciences, University of Milan, Via Commenda 10, 20122 Milan, Italy

**Keywords:** hydrogen, zebrafish, osteoporosis, antioxidant

## Abstract

Antioxidants represent a powerful tool for many human diseases and, in particular, molecular hydrogen has unique characteristics that make it a very promising therapeutic agent against osteoporosis. Zebrafish scales offer an innovative model in which new therapeutic approaches against secondary osteoporosis are tested. Scale bone loss obtained by prednisolone (PN) treatment is characterized by increased osteoclast activity and decreased osteoblast activity highlighted with bone enzymatic assays. We used this read-out system to test the therapeutic effects of hydrogen-rich water (HRW), an innovative antioxidant approach. HRW prevented osteoclast activation and bone loss in PN-treated fish scales, as verified by both biochemical and histochemical tartrate-resistant alkaline phosphatase assays. On the other hand, HRW treatment did not prevent PN-dependent osteoblast suppression, as measured by alkaline phosphatase activity. Moreover, HRW treatment did not facilitate the reparation of resorption lacunae induced in scales by PN. Our study highlighted a specific effect of HRW on adult osteoclast activity but not in osteoblasts, introducing an intriguing new antioxidant preventive approach against osteoporosis.

## 1. Introduction

Osteoporosis is a chronic bone disease characterized by a high imbalance between osteoclast and osteoblast functions that leads to increased bone resorption and decreased bone matrix deposition, low bone mass and altered microarchitecture of bone tissue. There are many factors that influence the pathogenesis of this disease, among which oxidative stress significantly influences bone health in both the generation and the progression of osteoporosis [1,2,3]. Reactive oxygen species (ROS) production is involved in bone homeostasis, acting on osteoblast and osteoclast differentiation [4] but also has a role in bone pathologies inducing osteoblast and osteocyte apoptosis while favoring osteoclastogenesis, thus inhibiting osteogenesis and promoting bone resorption [5]. Drugs and treatments currently used for the treatment of osteoporosis in humans show many important side effects [6]; this makes it necessary and a priority to find less invasive therapeutic approaches with as few side effects as possible. In this regard, hydrogen rich water (HRW) represents a captivating, efficient, safe and non-invasive therapeutic approach [7]. HRW, with its high content of molecular hydrogen (H_2_) gas has amazing reducing and antioxidant properties and many advantages compared to the most commonly used antioxidants, such as vitamin C and E [8]. In fact, H_2_ has the unique characteristic of not reacting with ROS with physiological roles but acting selectively by reducing cytotoxic ROS, such as the hydroxyl radical. Moreover, H_2_ is a very small molecule that is also electrically neutral, so it can easily penetrate cellular membranes and quickly diffuse into the cytosol, carrying antioxidant activity up to the nucleus and mitochondria, where other antioxidants cannot reach [9]. H_2_ does not show any toxicity, even at high concentrations [10]. All these characteristics make H_2_ a very promising therapeutic agent, used both in preventive and curative protocols, against a variety of diseases where oxidative stress has a dominant role, such as non-alcoholic fatty liver disease [11], cancer [12], COVID-19 complications [13] and neurodegenerative disorders [14]. 

Hydrogen-rich water (HRW) has been used in a variety of in vitro and in vivo models to take advantage of its antioxidant activity and to date, some clinical applications have also been approved [15]. 

Concerning bone studies, few data have been produced in recent years using molecular hydrogen. Li et al. showed in vitro effects of H_2_ treatment on murine RAW264.7 cells in the prevention of RANKL-induced osteoclast differentiation associated with reduction of the expression of osteoclast-specific mRNA markers and with inhibition of ROS generation [16]. Moreover, another in vitro study concerning H_2_ effects on osteoblasts was conducted on TNFα-induced injury cultured rat osteoblasts characterized by inhibition of alkaline phosphatase (ALP) activity and Runx2 mRNA expression. H_2_ incubation reversed these alterations, alleviating cell injury by decreasing oxidative stress and inflammation and preserving mitochondrial functions [17].

Some in vivo studies have been performed concerning the effects of HRW in pathological bone models. The first demonstration of the protective effect of H_2_ on bone tissue in vivo has been performed against microgravity–induced bone loss in a rat model with reduction of microgravity-induced ROS formation, osteoclastogenesis and osteoclast differentiation [18]. Another study by Guo et al. investigated HRW effects on ovariectomy-induced osteoporosis in rats, showing that its consumption prevents osteopenia, possibly through the ablation of oxidative stress induced by estrogen withdrawal [19]. 

In human studies, molecular hydrogen, administrated together with conventional therapy, has demonstrated great effects on the symptoms of patients with arthritis rheumatoid, especially in the early phase [20].

Interestingly, H_2_ reduced oxidative stress, as demonstrated by 8-deoxy-guanosine (8-OHdG) measurement and extended the effect (up to 4 weeks) after suspension of the administration, indicating a potential effect on long-term enzymatic expression or activity [20]. Another clinical study on rheumatoid arthritis patients was performed using H_2_-rich saline infusion and disease activity, inflammatory cytokines and disease markers were evaluated in the blood. After 4 weeks, in the joints, the disease activity decreased together with 8-OHdG, IL-6 and MMP-3 levels, indicating a reduction of oxidative stress and inflammatory conditions [21].

**Figure 1:**
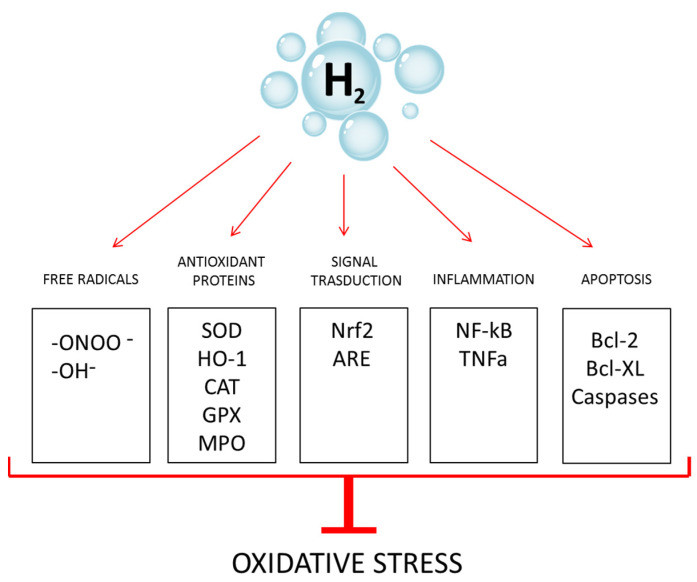
Molecular hydrogen counteracts oxidative stress through multiple pathways. -OH^−^: hydroxyl radical; -ONOO^-^: peroxynitrite anion; SOD: superoxide dismutase; CAT: catalase, MPO: myeloperoxidase; HO-1: heme oxygenase; ARE: antioxidant responsive element; GPX: glutathione peroxidase.

Many studies have shown that hydrogen water has antioxidant and anti-inflammatory effects; nevertheless, its molecular mechanism remains largely unclear, and some substrates and pathways have been identified (Figure 1).

The main target of the antioxidant activity of H_2_ seems to be hydroxyl radicals, as demonstrated in vitro in the suppression of mouse embryonic fibroblast senescence [22] and in the protection of radiation-derived cellular damage in male germ cells [23].

The hydroxyl radical is strongly involved in lipid peroxidation and in the modification of deoxyguanosine to 8-hydroxyguanosine, which are very dangerous for membranes and DNA integrity, respectively. 

Another direct target of hydrogen is the nitrification of tyrosine on proteins. A clinical study demonstrated that drinking hydrogen water is able to reduce the level of nitrotyrosines in patients with rheumatoid arthritis [20]. On the contrary, hydrogen peroxide cannot be directly modulated by molecular hydrogen [24].

The antioxidant activity of hydrogen also seems to act indirectly by regulating protein activity and gene expression. In fact, it is able to suppress the activity of NADPH oxidase and upregulate the gene expression of catalase, superoxide dismutase, HO-1 and myeloperoxidase [24]. One of the most important molecular messengers of hydrogen inside the cell is Nuclear factor erythroid-related factor 2 (Nrf2), which modulates the transcription of several genes involved in the endogenous antioxidant system [25]. The hydrogen-mediated regulation of gene expression also includes the suppression of inflammatory cytokines and their regulators, such as NF-kB, tumor necrosis factor alpha (TNFa), interleukin-1 (IL-1), interleukin-6 (IL-6), interleukin-12 (IL-12), C-C motif chemokine ligand 21, interferon gamma, prostaglandin E2 and high mobility group box-1 [26]. In addition, it has been demonstrated that hydrogen stimulates the anti-apoptotic pathway by modulating the expression of caspases and Bcl-2 family proteins [24] and the adrenal receptor pathway, as seen in neuroprotection of Parkinson’s disease patients [27].

*Danio rerio* (zebrafish) is an innovative and powerful model for the study of bone diseases [28,29]. In particular, an adult zebrafish model of glucocorticoid-induced osteoporosis (GIOP) has been created by prednisolone (PN) treatment that induces dose-dependent mineral matrix loss in fish scales. The phenotype of the GIOP scale resembles the characteristics of the human osteoporotic bone, with reduced mineral content, presence of resorption lacunae and alteration of bone markers such as increased tartrate resistant acid phosphatase (TRAP) activity and reduced ALP activity [30]. 

Only a few studies with H_2_ have been performed so far in zebrafish models, mostly concerning its anti-inflammatory properties in models of viral [31] and bacterial [32] infections. To date, only one study has been published concerning the effects of HRW and bone metabolism; our study focuses on the ability of molecular hydrogen to enhance embryo osteogenesis [33]. The aim of this study was to evaluate the effects of HRW in adult zebrafish bone tissue and, in particular, its ability to prevent the formation of the osteoporotic phenotype in zebrafish GIOP scales. Due to the characteristics of accessibility and transparency, the scale could represent an elective model for further dissecting the molecular mechanisms of hydrogen antioxidant activity in adult bone metabolism.

## 2. Materials and Methods

### 2.1. Ethic Statement

This experiment was performed in the Zebrafish Laboratory (IRCCS R. Galeazzi, GSD Foundation, Milan, Italy) according to European and Italian guidelines on animal research (EU Directive 2010/63/EU). Zebrafish experimentation and all the protocols of this study were approved by the Italian Ministry of Health, with authorization n. 805/2021-PR of 10/19/2021.

### 2.2. Animals and Treatments 

Ten-month-old AB strain zebrafish were maintained in ZEBTEC© Bench Top System (Tecniplast, Buguggiate, Italy) under standard conditions [34] at 28 °C. GIOP fish were treated following our previously published protocol [30] with prednisolone (Dehydrocortisone, PN, Sigma Aldrich, St. Louis, MO, USA) initially dissolved in dimethyl sulfoxide (DMSO, Sigma Aldrich) and then diluted in ZEBTEC© fish water (FW) to a final concentration of 80 µM. Moreover, other fish were treated with 80µM PN for 14 days and then treated for another 14 days in FW with/without HRW to verify the HRW curative effects in the GIOP model. A hydrogen generator bottle (H10 Gomax) was used to generate hydrogen-enriched water starting from FW, thanks to the water electrolysis process that produces molecular hydrogen. This water was diluted in FW (1:13 *v*/*v*) to make the HRW used for the treatments equal to 7.7% (pH 7.6). This final concentration was determined in our previously published study [33] as the biologically active and non-toxic concentration whose treatment on zebrafish embryos induces a statistically significant increase in vertebral mineralization. Experiments were performed with 14-day treatments through direct immersion of fish in FW or HRW with/without PN changing the treatment solution two times each day.

### 2.3. Oxidation–Reduction Potential Measurements

The oxidation–reduction potential (ORP) was measured in order to determine the oxidizing or reducing potential of a water sample. It was measured by the electrodes of an ORP meter; a positive ORP value means that the water is oxidative, while a negative value indicates that the water is reducing. ORP was measured with an ORP-meter (ORP-969, Seafront, Beijing, China). Every measurement was performed using the same amount of water, and the same fish tank in which all the treatments of this experimentation were also performed. That is important because ORP is closely related to the presence of free molecular hydrogen in the water and the hydrogen tends to quickly disperse in the air, so the use of the same tank guarantees the same air–water contact surface. ORP measurements were performed right after the withdrawal of FW from the ZEBTEC© system, and the creation and dilution of HRW were repeated initially every 15 min then at more spaced intervals up to 160 min as ORP levels reached equilibrium.

### 2.4. Scale Collection

At the end of the treatments, the fish were washed in E3 medium solution and then anesthetized using 0.16 mg/mL tricaine methane sulfonate solution [34]. E3 medium solution was prepared to have final concentrations of 5 mM NaCl, 0.17 mM KCl, 0.33 mM CaCl_2_ and 0.33 mM MgSO_4_. The scales were carefully removed from both sides of fish bodies using Dumont^®^ stainless steel forceps (Sigma Aldrich, St. Louis, MO, USA) operating under a stereomicroscope (Olympus SZX-ZB7, Tokyo, Japan).

### 2.5. Histological TRAP and ALP Assays in Scales

Histological TRAP activity was evaluated on explanted scales using a leukocyte acid phosphatase (TRAP) detection kit (Sigma Aldrich) processing scales according to the manufacturer’s protocol. Using TRAP staining images acquired using a stereomicroscope (Olympus SZX-ZB7) equipped with a Blacklight5000 camera (TiEsseLab, Milan, Italy), it was possible to quantify the percentage of mineral matrix resorption using ISC Capture Software (TiEsseLab). 

Histological ALP activity was detected on explanted scales fixed in 10% formalin and 0.1 M sodium phosphate solution (pH 7.4) and then exposed to BCIP^®^/NBT liquid ready to use substrate (Sigma Aldrich) according to the manufacturer’s protocol. After staining, the scales were mounted on glass microscope slides, and images were acquired using a stereomicroscope (Olympus SZX-ZB7) equipped with a Blacklight5000 camera (TiEsseLab). 

### 2.6. Biochemical TRAP and ALP Assays in Scales

Biochemical TRAP and ALP activity were performed directly on explanted scales. Specific osteoblast ALP activity was biochemically detected according to our previously published method [35]. Briefly, 20 scales for each fish were fixed in 10% formalin and 0.1 M sodium phosphate solution (pH 7.4), then washed in demineralized water and cut with a scalpel in order to use only the epidermis-free half-scale part to avoid the interference of the high intensity ALP signal of the blood vessels present in the half scale covered by epidermis. Each epidermis-free half-scale was singularly incubated with 450 µL of alkaline buffer (100 mM TRIS HCL, 1 mM MgCl_2_, 0.1 mM ZnCl_2_ pH 9.5) for 30 min and then incubated with 150 µL of alkaline buffer with 20 mM pNPP (4-nitrophenyl phosphate disodium salt hexahydrate, Sigma). After 1 h, the reaction was stopped using a 3N NaOH 20 mM EDTA solution. The supernatant solution was transferred in a new plate, and its absorbance was evaluated at 405 nm using a spectrophotometer (iMarkTM Microplate Reader, Bio-Rad, Hercules, CA, USA).

A biochemical TRAP assay was performed on non-cut scales following the protocol of Perrson et al. [36]. Briefly, scales were fixed as previously described for the biochemical ALP assay and then incubated in 0.1 M sodium acetate and 20 mM tartrate buffer (pH 5.3) for 1 h. Each scale was incubated with 150 µL of 0.1 M sodium acetate, 20 mM tartrate and 20 mM pNPP for 3 h. Then, the reaction was stopped by adding 50 µL of 2N NaOH, and the supernatant solution was transferred to a new plate and spectrophotometrically assayed at 405 nm (iMarkTM Microplate Reader, Bio-Rad).

### 2.7. Double Bone Matrix Vital Staining

Evaluation of the bone matrix profile was performed by direct immersion of the fish in two different staining solutions, the first with alizarin red S, to stain the mineralized matrix present in the scales of the GIOP fish model, and the second with calcein, to highlight the new mineralized matrix deposed during the healing process that closes the resorption lacunae in the scale borders. In detail, fish treated with PN and then used to test HRW healing properties were stained overnight in the dark with 0.005 alizarin red S (Sigma) pH 7.5 E3 solution after 14 days of treatment. After another 14 days with/without HRW, a second staining was performed using 0.005% calcein (Sigma) pH 7.5 solution overnight in the dark. Then, fish were repeatedly washed with E3 medium solution, and scales were explanted, as described before, fixed using 3.4% formaldehyde 0.1M sodium phosphate buffer solution for 15 min and then analyzed using a fluorescence microscope (Olympus SZX-ZB7). Merging the images using ISC Capture Software (TiEsseLab) with red alizarin red S staining and the green signal of calcein staining, it was possible to highlight the new mineralized matrix of the closing resorption lacunae along the scale borders.

### 2.8. Statistics

In each experiment, three fish were used for each treatment group, with a total of 54 fish. Data derived from biochemical analysis of ALP and TRAP activity in adult scales were obtained by testing 20 scales for fish, and results are expressed as mean ± standard deviation versus control. Histological analysis was also performed on 20 scales for fish. Data from each tested fish age were subjected to variance analysis with one-way Fisher’s test followed by Student’s *t*-test using SigmaStat 3.5 Software. All significance values were set at *p* < 0.05 (*), *p* < 0.01 (**) and *p* < 0.001 (***), and all experiments were repeated at least three times with comparable results.

## 3. Results

### 3.1. ORP Measurements

**Figure 2:**
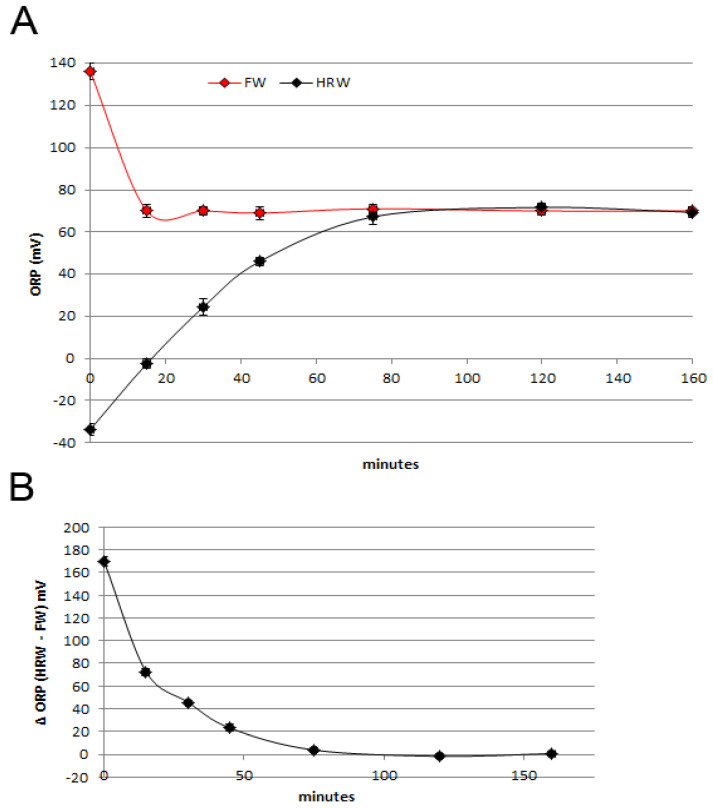
(**A**) ORP measurements performed on FW and HRW right after production: dilution of HRW and withdrawal of FW from the ZEBTEC© system. FW ORP quickly drops from about 140 mV to a stable level of about 70 mV. (**B**) ORP difference between FW and HRW expressed as ΔORP (HRW ORP − FW ORP, mV), which reaches 0 after about 75 min.

We measured the ORP (Figure 2A) of a fresh FW sample, finding a mean initial level of 135 mV that quickly decreased up to a mean of 70 mV, which was stable over time. This depends on the rapid balancing of the water temperature with the environment one (from 27.5 °C of FW to approximately 24.0 °C of the room) since temperature has a direct effect on the ORP value. HRW was created by dilution of hydrogen-enriched water generated with the hydrogen generator bottle in FW (1:13 *v*/*v*; pH 7.6). The starting ORP level, measured immediately after the dilution, was about −40 mV, and then the ORP value slowly increased for approximately 75 min until the plateau reached 70 mV, in equilibrium with the FW sample. The ORP difference between FW and HRW was evaluated as HRW ORP - FW ORP and visualized in Figure 2B, highlighting the decreasing difference up to the 0 value around 90 min.

### 3.2. HRW Treatment Prevents Osteoclast Activation in PN-Treated Fish Scales

**Figure 3:**
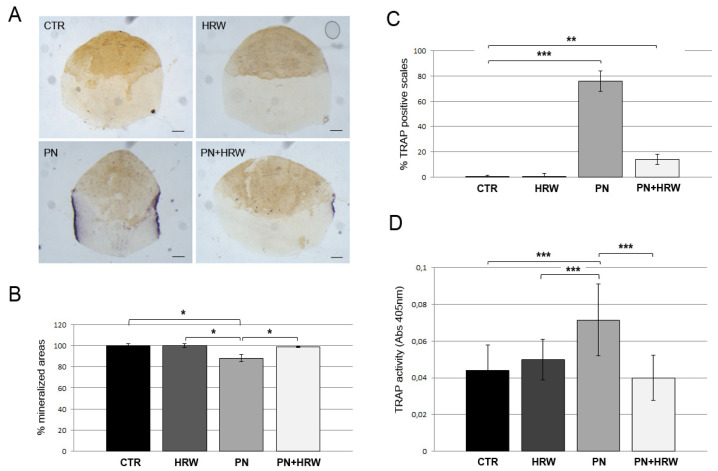
(**A**) Histological TRAP activity assay highlighted by violet staining of enzymatic activity along the scale borders of PN and PN + HRW scales. Scale bar 0.22 mm. (**B**) Percentage of mineralized area of scales with PN scales area reduced by 11.8% (PN vs. CTR, *p* < 0.05 *; PN vs. HRW, *p* < 0.05 *; PN vs. PN+HRW, *p* < 0.05 *). (**C**) Percentage of TRAP positive scales equal to 76% in PN fish and 14% in PN + HRW fish (CTR vs. PN, *p* < 0.001 ***; CTR vs. PN+HRW, *p* < 0.01 **). (**D**) Biochemical TRAP assay shows increased TRAP levels only in PN fish scales (PN vs. CTR, *p* < 0.001 ***; PN vs. HRW, *p* < 0.001 ***; PN vs. PN+HRW, *p* < 0.001 ***).

First, we measured the ability of HRW to prevent PN-induced bone loss in zebrafish scales. To this purpose, we added HRW and PN alone or together, and we evaluated the generation of the bone loss phenotype (Figure 5A,B, PREVENTIVE MODE). The osteoclast resorption activity in fish scales was evaluated by a histochemical TRAP assay. HRW fish scales did not show any TRAP staining since there was no osteoclast activation, as in CTR scales. PN treatment induced osteoclast activity along scale borders, which was highly reduced by HRW co-treatment (Figure 3A). In fact, 76% PN scales were positive for TRAP staining (Figure 3C), and the mineralized area of the scales was reduced 11.8% by osteoclast resorption, while PN + HRW scales showed TRAP staining only in 14% scales with a not statistically significant reduction of scale area equal to 1.3% (Figure 3B). Biochemical assays performed on explanted scales confirmed the results of histological tests. PN treatment induced an increase of 61.7% in scale TRAP activity, whereas HRW co-treatment inhibited its effect (Figure 3D).

### 3.3. HRW Treatment Does Not Prevent PN-Dependent Downregulation of ALP Activity in Scale Osteoblasts

**Figure 4:**
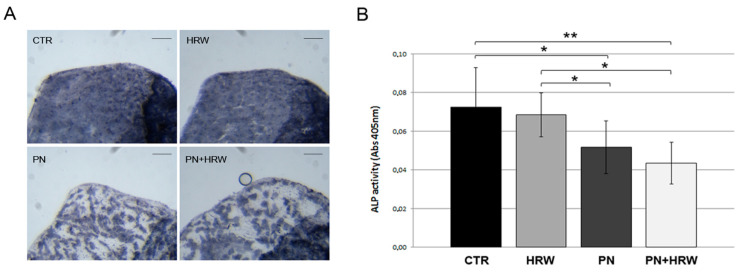
(**A**) Histochemical ALP activity assay highlighted by blue staining of the localization of enzymatic activity in the scale. CTR and HRW scales show normal ALP activity, while PN and PN + HRW scales show decreased ALP activity, visualized as white unstained areas. Scale bar 0.11 mm. (**B**) Quantification of ALP activity performed with biochemical assay shows decreased ALP activity on PN and PN + HRW scales (PN vs. CTR, *p* < 0.05 *; PN vs. HRW, *p* < 0.05 *; PN + HRW vs. CTR, *p* < 0.01 **; PN + HRW vs. HRW, *p* < 0.05 *).

HRW treatment did not modulate osteoblast functions, and ALP staining was uniform throughout the scale as in CTR scales. PN treatment affected zebrafish scale osteoblast function, downregulating the ALP enzymatic activity that results in unstained areas, whereas HRW co-treatment did not prevent PN-induced alteration in ALP activity distribution (Figure 4A). Regarding quantitative data, PN induced a decrease of 28.5% in ALP activity, but HRW co-treatment was not able to prevent the effect (Figure 4B). As controls, HRW alone did not induce statistically significant modulation in either enzymatic activity levels.

### 3.4. HRW Does Not Facilitate Bone Tissue Repair in Scales after PN Treatment 

**Figure 5:**
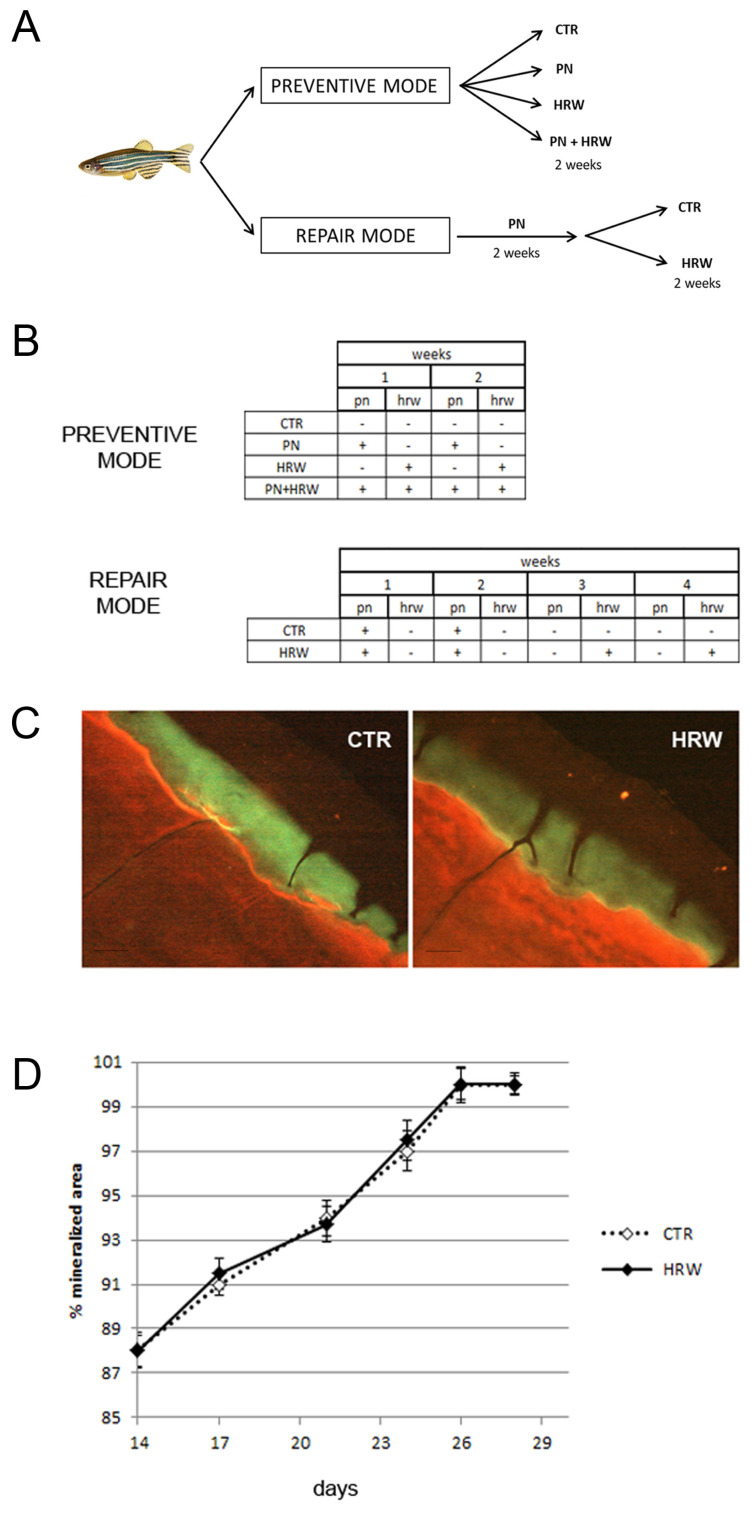
(**A**) Schematic representation of the experiment performed in PREVENTIVE MODE and REPAIR MODE with CTR, PN, HRW and PN + HRW samples (CTR: untreated, PN: treated with prednisolone, HRW: treated with hydrogen water, PN+HRW: treated with hydrogen water and prednisolone). (**B**) Prednisolone (pn) and hydrogen rich water (hrw) were administrated with different methods and timing in CTR, PN, HRW and PN + HRW samples in PREVENTIVE MODE or REPAIR MODE. (**C**) Exemplificative picture of the reparative process in resorption areas of HRW or untreated CTR fish after prednisolone treatment (REPAIR MODE). Green fluorescence indicates a new mineralized matrix deposed in the lacuna. Scale bar 0.02 mm. (**D**) % of scale mineralized area measured in 3rd and 4th weeks in HRW and untreated CTR fish after PN treatment (REPAIR MODE). The reparative process was not modulated by the HRW treatment.

Next, we measured the ability of HRW to stimulate the repair of bone resorption lacunae generated by prednisolone. To this purpose, we added HRW to the fish water after two weeks of PN treatment, and we let the resorption areas get filled for the next two weeks (Figure 5A,B, REPAIR MODE). The filled areas in controls and HRW fish were visualized in green fluorescence by calcein-Alizarin red double staining (Figure 5C). The measure of lacuna areas in the time course indicated that HRW treatment did not facilitate the filling process until full repair (Figure 5D).

## 4. Discussion

Oxidative stress alters bone remodeling by affecting osteoblast activity and by enhancing osteoclast activity, contributing to the development of many bone diseases, the most important of which is osteoporosis, characterized by low bone mineral density and decreased bone mass [5]. ROS are fundamental components in the regulation of bone homeostasis and osteoclast differentiation, proliferation and survival [4]. Under physiological conditions, osteoclasts produce ROS themselves to enhance bone tissue resorption, but ROS also play an important role in the pathogenesis of bone diseases, inducing abnormal apoptosis of osteocytes and osteoblasts while promoting osteoclast activity [2]. 

Antioxidants have great effects on bone tissue, activating osteoblast differentiation, promoting bone formation and also reducing osteoclast resorption activity [5,37]. Targeting ROS with antioxidant compounds could lead to the development of novel therapeutic strategies for bone diseases and HW represents an intriguing new non-invasive and non-toxic approach [6].

Adult zebrafish offers the scale model as an innovative read-out system for adult bone tissue studies; in particular, the GIOP model represents a fast and powerful resource to study osteoporotic scale metabolism and new therapeutic approaches. The zebrafish GIOP model is characterized by increased osteoclast activity and decreased osteoblast activity in scales that can be highlighted using both histological and biochemical ALP and TRAP activity assays. We used this model to test the therapeutic effects of HRW, highlighting a specific effect on osteoclast activity but not on osteoblast activity. First, we performed ORP measurements of fish treatment water to identify the reducing potential of HRW and the effective treatment time for the fish. HRW treatment was effective on osteoclast activity. 

H_2_ effects on osteoclasts have been the object of many in vitro and in vivo studies. An in vitro study performed on RAW264.7 murine cells showed that H_2_ treatment prevents RANKL-associated osteoclastogenesis, inhibiting the inactivation of NF-kB, mitogen-activated protein kinase (MAPK) and AKT pathways. H_2_ treatment also reduces the expression of specific osteoclast markers, such as TRAP, calcitonin receptor, cathepsin K, and intracellular and mitochondrial ROS formation [16]. Concerning in vivo studies, the first and the most important experimentation demonstrated the protective activity of H_2_ on microgravity–induced bone loss in a rat model with the reduction of ROS formation and even osteoclastogenesis [17]. Another study that confirmed the in vivo H_2_ effects showed the HW ability to prevent the negative effects of ovariectomy in a rat model, avoiding the development of an osteoporotic state [18].

ROS activity is crucial in osteoclast differentiation because of the regulation of mitogen-activated protein kinases, intracellular calcium levels and several transcription factors such as NF-κB [38]. During osteoclast differentiation, the expression of endogenous antioxidant enzymes is also decreased [39]. It is also known that glucocorticoids, such as prednisolone, promote osteoclastogenesis by upregulating ROS levels [40]. Interestingly, glucocorticoids are able to suppress the expression of Nrf2 [41,42], a key transcription factor that stimulates the synthesis of several antioxidant proteins such as OH-1 and is found to be a potential pharmacological target to block osteoclastogenesis [43]. 

Considering these data, we suggest that in the zebrafish GIOP model, PPN induces osteoclast differentiation by increasing ROS and suppressing Nrf2-dependent pathways. Molecular hydrogen administration scavenges ROS and stimulates Nrf2 in pre-osteoclasts, preventing osteoclast activation (PREVENTIVE MODE). Once osteoclasts are active in the resorbing phase, they cannot be switched off by redox modulation (REPAIR MODE). 

Our study, on the contrary, indicated that HRW treatment does not influence the PN-induced suppression of osteoblast functions in co-administration (PREVENTIVE MODE) and does not modulate the efficiency of osteoblast deposition to repair the resorption lacunae generated by prednisolone (REPAIR MODE). An in vitro study on injured rat osteoblasts has shown that H_2_ protects ALP activity from TNFα-induced down-regulation [17], whereas in vivo, osteoblast mineralizing activity can be stimulated by hydrogen in physiological conditions, as demonstrated in our laboratory during embryonic development in zebrafish [33]. In inflammatory and developmental conditions, redox regulation could play a fundamental role [44]. Nevertheless, PN may affect different pathways in vivo in adult zebrafish during scale bone remodeling and is not related to ROS. In fact, many factors have been studied and found to be stimulatory for osteoblast differentiation and activity, such as bone morphogenic proteins (BMPs), Wnt pathway, cytokines and growth factors [45]. For example, it is known that glucocorticoids suppress osteoblastic Wnt16 expression in vitro and in vivo and, consequently, ALP activity [46]. 

Our results suggested that the reduced ALP activity in osteoblasts after PN treatment in scales was not strictly dependent on redox mechanisms. In this condition, hydrogen cannot counteract the PN effects by its antioxidant activity. In addition, osteoblast activation in the reparative process is not sufficiently modulated by oxidative stress signals. 

In conclusion, HRW shows in vivo anti-osteoporotic activity on an adult zebrafish scale GIOP model preventing osteoclast activation but does not contrast with the PN-induced reduction of osteoblast activity. The adult zebrafish model represents an elective model for dissecting the molecular mechanisms of the antioxidant activity of hydrogen in vivo. In addition, HRW has been confirmed as an interesting new preventive approach against osteoporosis.

## Data Availability

Data are contained within the article.
